# Supplementary material for: Optimal construction of a functional interaction network from pooled library CRISPR fitness screens
Source: BMC Bioinformatics. 2022 Nov 28;23:510. doi: 10.1186/s12859-022-05078-y (PMC9707256; doi:10.1186/s12859-022-05078-y)
Supplement: Supplementary file 1 — Additional file 1. Figure S1. Boyle PCA as variance normalization. A Bar plot showing the percentage of variance explained by each principal component of the Boyle PCA approach across Olfactory receptor genes, applied to the Z-score data matrix. B Scatter plot of the standard deviation of the screen, using Z-scores data matrix, versus the screen-wise projection onto the first Principal component from the Boyle approach. Figure S2. LLS with other reference sets. A Ceres+PCC network evaluated using 3 different reference sets in the Log-likelihood analysis. Cumulative LL Scores and number of co-functional interactions in bins are plotted for the network, evaluated with Kegg, Reactome and GO reference sets. B Pathways containing genes associated with mitochondrial translation and oxidative phosphorylation were removed from Reactome and GO, to create CleanReactome and CleanGO. Cumulative LL Scores and number of co-functional interactions in bins are plotted for the Ceres+PCC network, evaluated with Kegg, Reactome and CleanReactome, GO and CleanGO reference sets. C Comparison of LLS scores and co-functional interactions of Ceres based networks using the full Reactome reference set and D the CleanReactome reference set in the LLS evaluation. Figure S3. Local LLS. A Local log-likelihood scores calculated per bin, using CleanReactome, for Ceres-based networks; B Chronos-based networks; C Bayes Factors based networks and D Z-scores based networks. Figure S4. Enrichment analysis for Ceres and Chronos based networks of equal size. A Venn diagrams depicting the numbers of genes and edges exclusive to the top 17k edges in the network created with Ceres+PCAwhitening+PCC versus the top 17k edges the network created with Chronos+PCAwhitening+PCC. B Enrichment of the gene set exclusive to Ceres+PCAwhitening+PCC. Only the top 30 enriched GO and Kegg terms are listed in the graph. C Enrichment of the gene set exclusive to Chronos+PCAwhitening+PCC. Figure S5. Pearson’s correlation coeff [file 12859_2022_5078_MOESM1_ESM.docx]

**Supplementary Figures:**

Figure S1

**A**


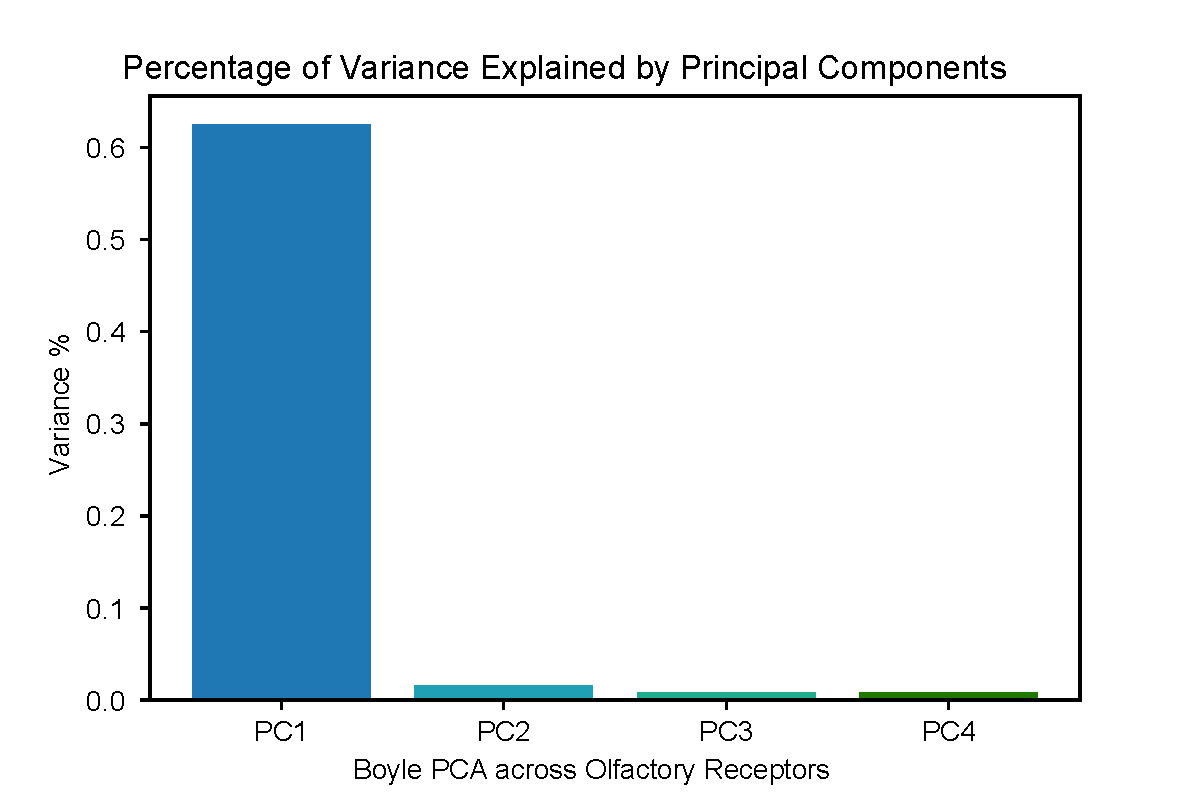


**B**


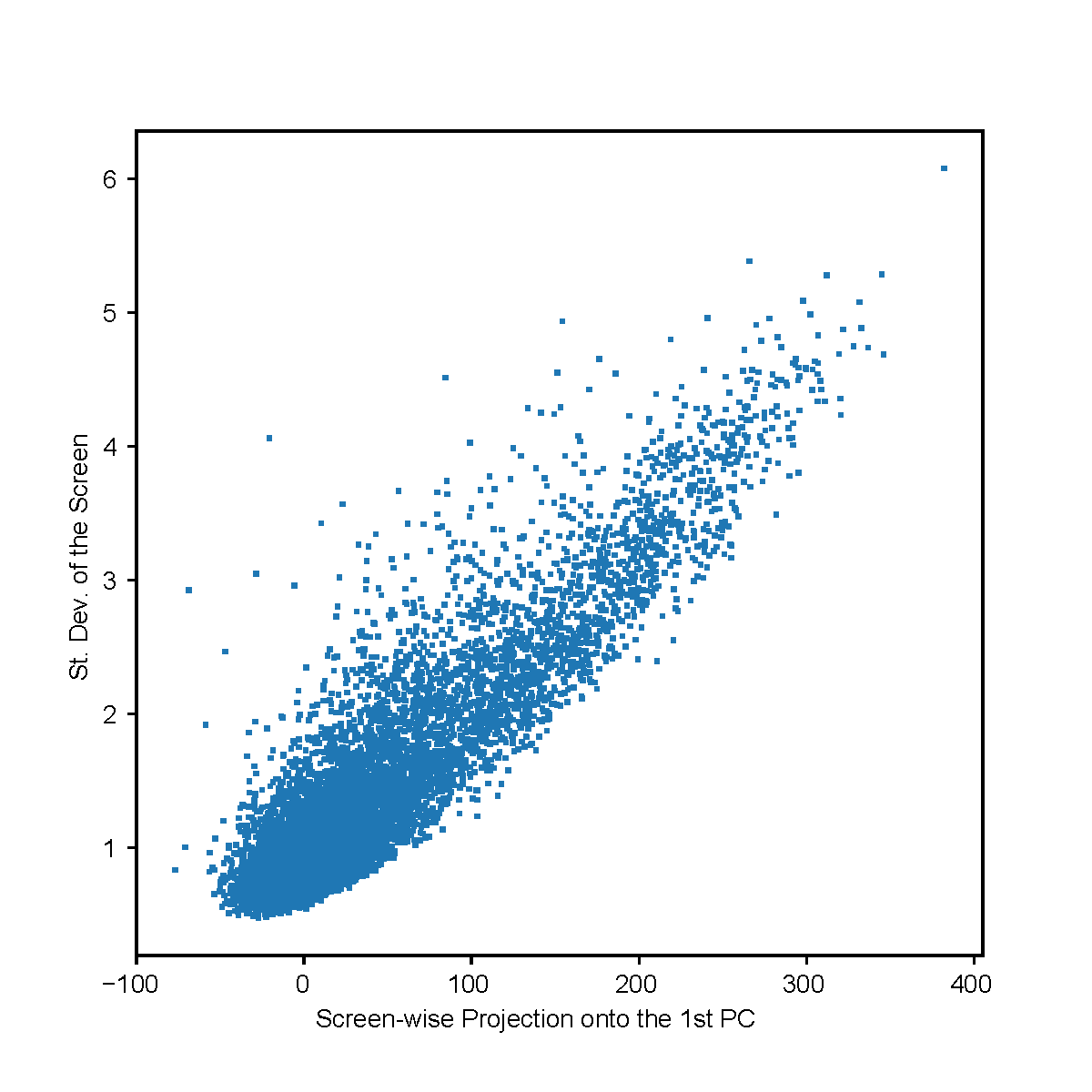


**Figure S1.** Boyle PCA as variance normalization. **A)** Bar plot showing the percentage of variance explained by each principal component of the Boyle PCA approach across Olfactory receptor genes, applied to the Z-score data matrix. **B)** Scatter plot of the standard deviation of the screen, using Z-scores data matrix, versus the screen-wise projection onto the first Principal component from the Boyle approach.

Figure S2


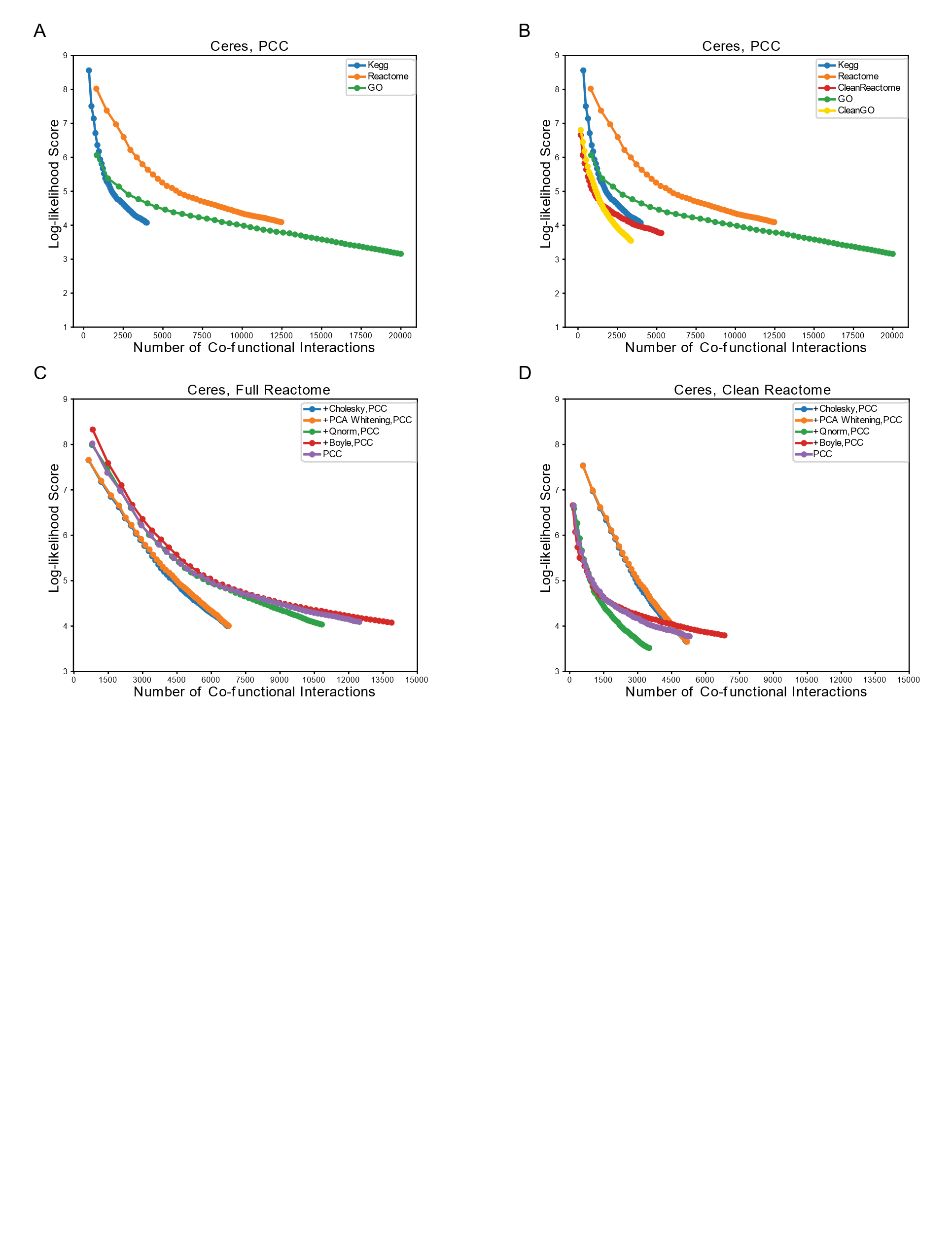


**Figure S2.** LLS with other reference sets. **A)** Ceres+PCC network evaluated using 3 different reference sets in the Log-likelihood analysis. Cumulative LL Scores and number of co-functional interactions in bins are plotted for the network, evaluated with Kegg, Reactome and GO reference sets. **B)** Pathways containing genes associated with mitochondrial translation and oxidative phosphorylation were removed from Reactome and GO, to create CleanReactome and CleanGO. Cumulative LL Scores and number of co-functional interactions in bins are plotted for the Ceres+PCC network, evaluated with Kegg, Reactome and CleanReactome, GO and CleanGO reference sets. **C)** Comparison of LLS scores and co-functional interactions of Ceres based networks using the full Reactome reference set and **D)** the CleanReactome reference set in the LLS evaluation.

Figure S3


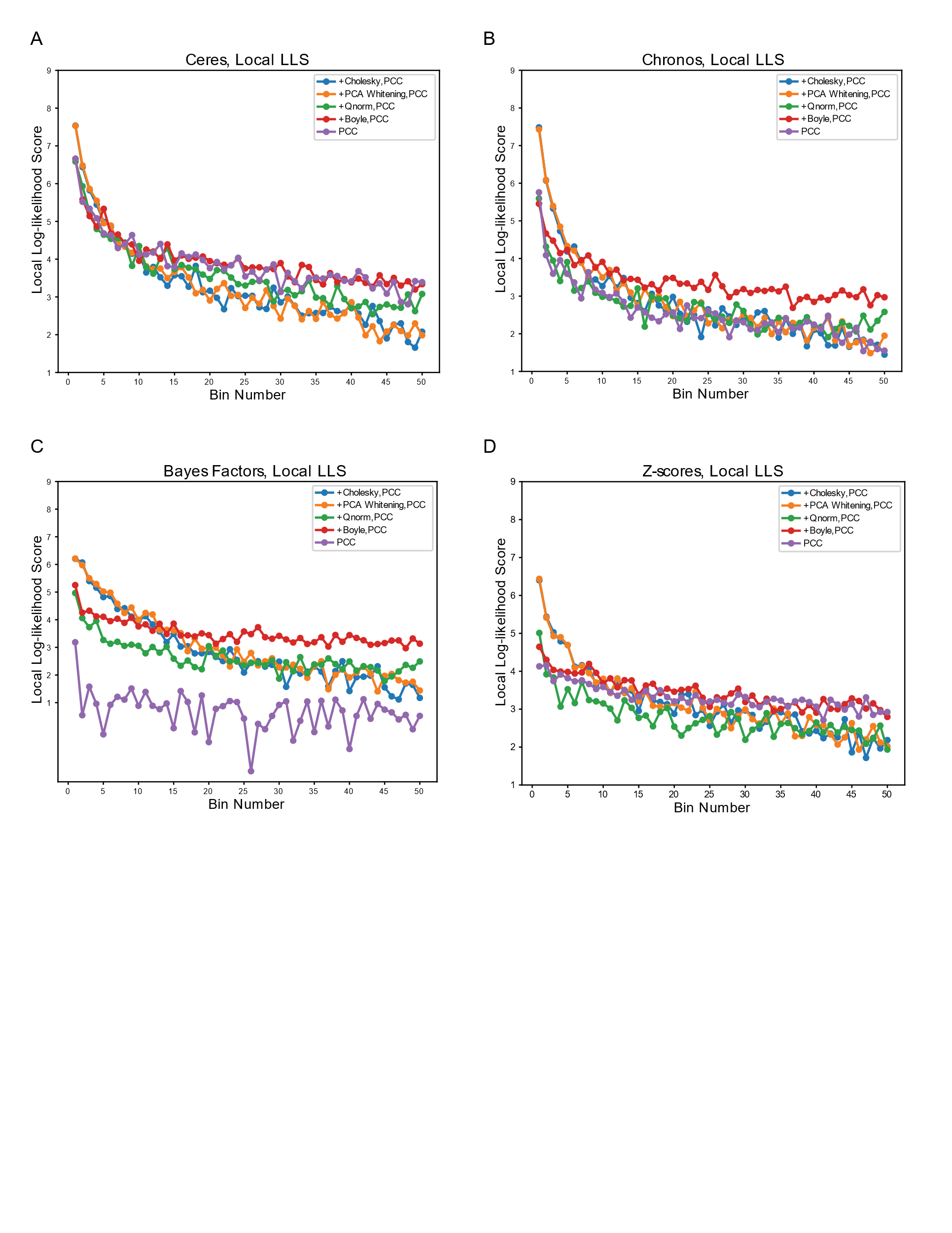


**Figure S3**. Local LLS. **A)** Local log-likelihood scores calculated per bin, using CleanReactome, for Ceres-based networks; **B)** Chronos-based networks; **C)** Bayes Factors based networks and **D)** Z-scores based networks.

Figure S4


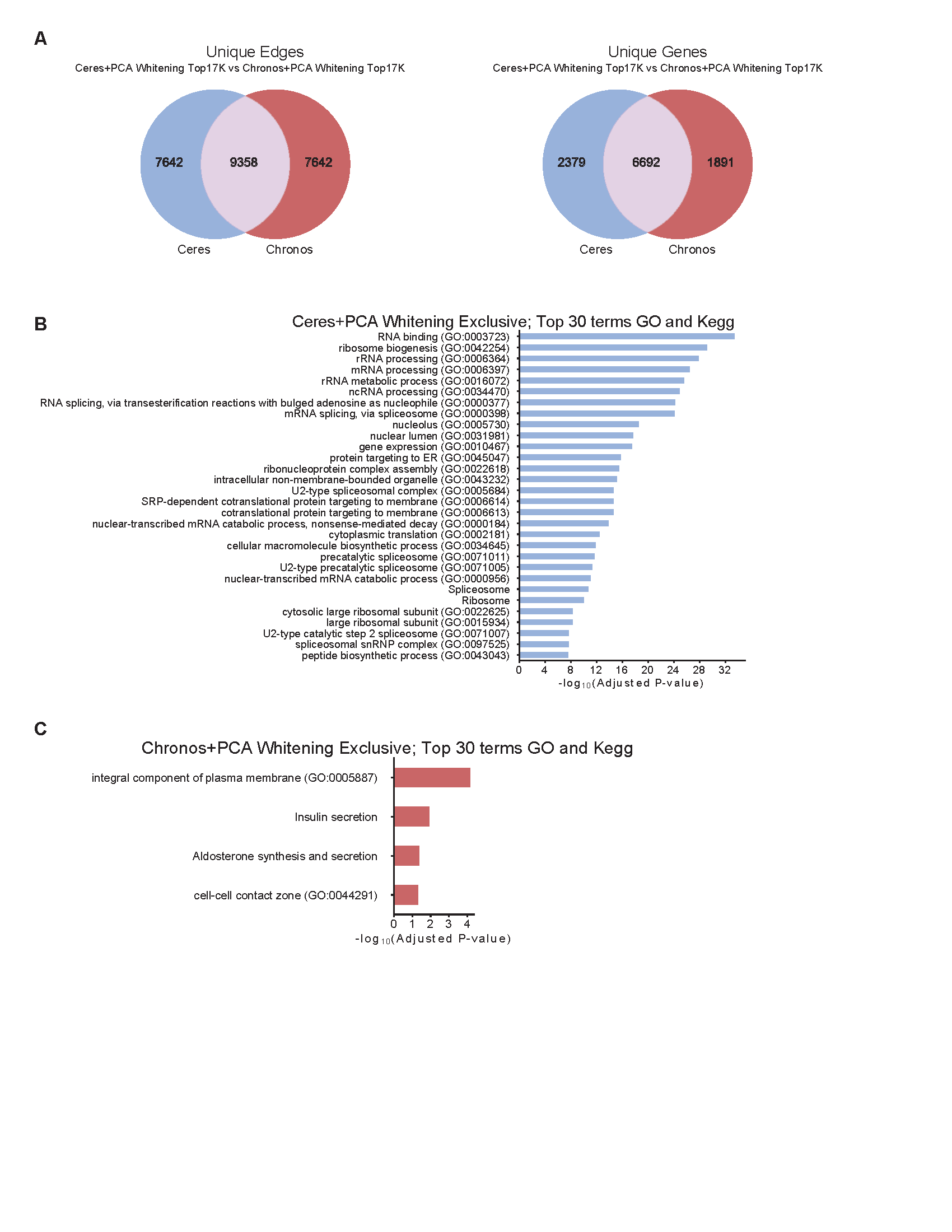


**Figure S4.** Enrichment analysis for Ceres and Chronos based networks of equal size. **A)** Venn diagrams depicting the numbers of genes and edges exclusive to the top 17k edges in the network created with Ceres+PCAwhitening+PCC vs. the top 17k edges the network created with Chronos+PCAwhitening+PCC. **B)** Enrichment of the gene set exclusive to Ceres+PCAwhitening+PCC. Only the top 30 enriched GO and Kegg terms are listed in the graph. **C)** Enrichment of the gene set exclusive to Chronos+PCAwhitening+PCC.

Figure S5


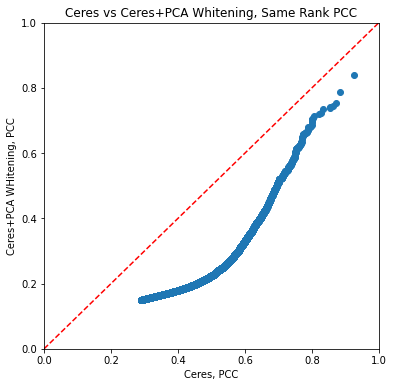


**Figure S5**. Pearson’s correlation coefficients of the edges of the same rank in the Ceres+PCA Whitening+PCC and Ceres+PCC networks.
